# Measuring the Ecological Footprint of Eating Behaviors: A Psychometric Study on the Turkish Version of the EREC Scale

**DOI:** 10.3390/nu18071132

**Published:** 2026-03-31

**Authors:** Busra Ayhan, Nazlıcan Erdogan Govez, Saniye Bilici, Eda Koksal, Nasminel Tekin

**Affiliations:** Department of Nutrition and Dietetics, Faculty of Health Sciences, Gazi University, Ankara 06490, Türkiye; nazlicanerdogan@gazi.edu.tr (N.E.G.); sgbilici@gazi.edu.tr (S.B.); edakoksal@gazi.edu.tr (E.K.); nasmineltekin@gazi.edu.tr (N.T.)

**Keywords:** eco-concern, eating behavior, validation, EREC, EDE-Q-13

## Abstract

Background/Objectives: This study was conducted to determine the validity and reliability of the Eating-Related Eco-Concern (EREC) in young Turkish adults and to evaluate the effect of ecological concerns on disordered eating characteristics, with a view to comparing these effects with the risk of eating disorders. Methods: The study included 600 young adults (138 males and 462 females) aged 18 to 35. Using face-to-face administration, the Eating Disorder Examination Questionnaire (EDE-Q-13) Short Form to assess eating disorder-related psychopathology and the EREC Scale to assess eating behaviors related to eco-concern were administered, and Turkish validity and reliability were examined. Results: The Kaiser–Meyer–Olkin (KMO) was 0.801, signifying acceptable sample adequacy, while Bartlett’s test of sphericity was significant (χ^2^ = 636.159, *p* < 0.001). All item factor loadings ranged from 0.582 to 0.767 and were statistically significant (*p* < 0.001). The scale’s Cronbach’s alpha was 0.854. Test–retest reliability was good, with an infraclass correlation coefficient (ICC) of 0.811 95% CI. The analysis revealed that the single-factor model demonstrated an acceptable fit to the data (χ^2^/*df* = 2.84, CFI = 0.976). There was no statistically significant correlation between EREC and the total EDE-Q-13 score (*p* = 0.064). On the other hand, the total EDE-Q-13 score was identified as a significant negative predictor of EREC scores (β = −2.648, *p* = 0.028). Conclusions: All item factors of the Turkish adaptation of the scale exhibit a structure that is quite consistent with the original scale. The 10-question version of EREC can be used with young adults in Türkiye. In this study, although ecological anxiety was associated with eating restraint or purging, it was not found to be generally associated with eating disorders.

## 1. Introduction

The American Psychological Association (APA) defines eco-concern as a persistent fear of environmental catastrophe [1]. Eco-anxiety refers to the psychological distress arising from environmental changes, particularly climate change, and encompasses a range of emotional responses, including anxiety, sadness, guilt, helplessness, frustration, and fear in relation to ecological degradation [2]. In contemporary media, the term eco-anxiety is more frequently used to describe youth and children, and it is particularly prevalent among young adults. There are concerns that young people’s eco-anxiety hinders their involvement in the environment and has a negative impact on their mental health [3,4]. A study of 10,000 young people found that 59% were very or extremely concerned about climate change, while 84% were at least moderately concerned. More than 45% of respondents reported that their daily functioning was negatively affected by their views on climate change [5]. Furthermore, eco-anxiety may make pre-existing mental health conditions worse or raise young people’s risk of mental illness [6]. A review of 12 studies found that eco-anxiety was associated with functional impairment, depression, anxiety, post-traumatic stress disorder, symptoms of stress and insomnia, low subjective mental health evaluation, and reluctance to have children [7].

Environmental concern has been linked to greater environmental awareness and, as such, may also be associated with the adoption of sustainable, health-conscious dietary behaviors [8]. Additionally, it has been reported that people’s choices for ecologically friendly cuisine may be positively correlated with their concerns about climate change [9]. In a study of young adults, those who adhered more closely to a Mediterranean diet had higher ecological concern scores [10]. Healthy eating habits have occasionally been found to be negatively connected with eco-anxiety. For instance, a study on college students found that higher levels of eco-anxiety were linked to lower levels of sustainable and healthful eating practices as well as reduced intake of organic food [8].

The literature also suggests that ecological concerns may influence risk factors associated with eating disorders. Climate change-related environmental concerns and insecurity with food may negatively affect eating disorder sufferers and might raise their likelihood of developing eating disorders [11]. Bulimic eating disorders were linked to mild eco-anxiety and emotional weariness, according to a study performed on university students [12]. In a similar vein, a case study revealed that a bulimic patient’s eco-anxiety was a major barrier to recovery and that the patient improved when it was treated as part of psychiatric treatment [13]. A study conducted on young women showed that eco-anxiety was directly proportional to orthorectic behavior [14].

However, little research has been conducted on the subject, and it remains unknown how environmental concerns affect eating habits. The Eating-Related Eco-Concern (EREC) Scale was developed by Qi et al. to assess how eco-concern affects adult eating patterns and to examine the relationship between eco-concern and eating disorders [2]. It is crucial to conduct validity and reliability research on the EREC Scale in the Turkish population because it is the first to assess the association between eco-concern and eating behaviors, and there are few studies on this topic. The hypotheses of this study are as follows: (a) The EREC scale is valid and reliable in young Turkish adults. (b) Ecological anxieties are positively associated with disordered eating characteristics and eating disorder behaviors. The purpose of this study was to assess the validity and reliability of the Eating-Related Eco-Concern (EREC) in young Turkish people, aas well as to identify the relationship between environmental anxieties and disordered eating characteristics.

## 2. Materials and Methods

### 2.1. Study Design and Participants

This methodological study was conducted between 20 March and April 2025, and 600 young adults (aged 18–35; 138 males, 462 females), who were randomly selected and volunteered to participate, were the subjects. To maintain an equal selection rate for each participant, a straightforward random sampling technique was employed. These young adults were enrolled at one of Türkiye’s largest universities, located in Ankara, Türkiye’s capital. A sample size of 50–100 participants was required for an exploratory factor analysis with 5–10 participants per scale item [15]. A sample size of 30–200 individuals was needed for confirmatory factor analysis, according to another study that recommended a minimum sample size of 3–20 times the number of variables in the scale [16]. Accordingly, the study aimed to reach over 200 participants, and the number was kept as high as possible.

Individuals were excluded from the study if they had a diagnosed psychological disorder or a chronic illness requiring a specific diet, were pregnant or breastfeeding, or did not provide informed consent to participate. Socio-demographic information, the Eating-Related Eco-Concern (EREC) Scale, and the Eating Disorder Examination Questionnaire (EDE-Q-13) Short Form were collected through face-to-face method questionnaire administration. The survey form used in the study is included as Supplementary Materials (File S1).

The Gazi University Ethics Committee approved the study (research code 2024-121, dated 9 January 2024). All subjects provided written informed consent, and the study was conducted in accordance with the tenets of the Declaration of Helsinki.

### 2.2. Scale Translation and Adaptation

For the Turkish validity and reliability analysis, permission was obtained via email from the original scale’s authors. The translation process adhered to the conventional procedure proposed by Brislin [17] and Prieto [18], employing a forward and back translation technique to ensure linguistic and conceptual consistency.

Following linguistic revisions, the scale underwent expert review, with opinions obtained from 10 specialists. In accordance with their recommendations, minor modifications were implemented. Finally, the revised scale was piloted with 20 eligible participants to gather feedback on item clarity and comprehension. The primary analysis did not incorporate data from this pilot trial.

### 2.3. Validation Process and Sample Size

**Figure 1:**
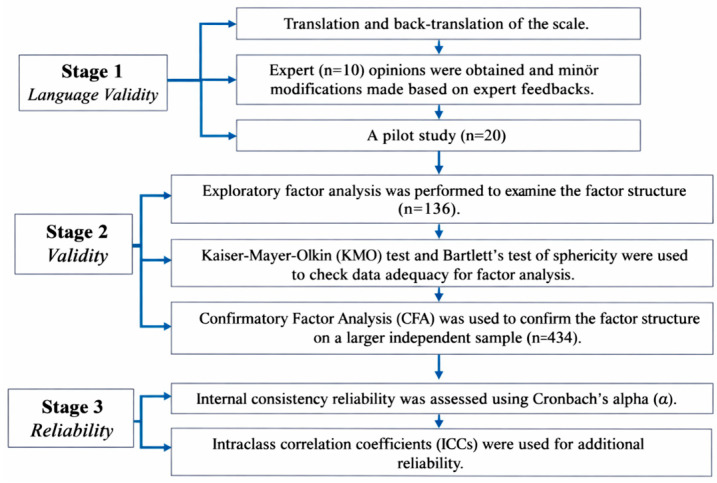
Flowchart of the study design.

First, an EFA was conducted on a smaller pilot sample to explore the scale’s factor structure. Second, confirmatory factor analysis (CFA), internal consistency reliability, and validity analyses were conducted on a larger, independent sample to test and confirm the structure. Importantly, these two stages involved entirely distinct participant groups with no overlap. It was intended to apply the original 10-item scale to at least 100 individuals in the first stage. The decision was based on the recommendation to establish a sample size for EFA in research conducted across several languages and/or cultures [19]. Figure 1 shows the study’s flowchart.

### 2.4. Eating-Related Eco-Concern (EREC) Scale

The EREC Scale, originally developed by Qi et al. [2], consists of 10 items designed to assess eco-concern-related eating behaviors. A 5-point Likert scale is used to rate the items, with 1 denoting “Never” and 5 denoting “Always.” A total score is calculated by summing the items, and higher scores indicate greater eco-concern in dietary behavior.

### 2.5. Eating Disorder Examination Questionnaire (EDE-Q-13) Short Form

The Eating Disorder Examination-Questionnaire (EDE-Q-13) Short Form is used to assess eating disorder-related psychopathology. The scale comprises four subscales: Restraint, Eating Concern, Shape Concern, and Weight Concern. It was initially developed by Lev-Ari et al. [20], and Esin and Ayyildiz [21] carried out the Turkish validity and reliability study. This short form was specifically preferred for its practicality, established Turkish validity, and utility in minimizing participant burden during data collection.

### 2.6. Validity, Reliability, and Statistical Analysis

IBM SPSS Statistics 29.0 and IBM SPSS Amos 22 were used for all statistical analyses. Internal consistency reliability was evaluated using the Cronbach’s alpha (α) coefficient. An α value ≥ 0.70 was accepted as the minimum threshold for acceptable reliability, with values ≥ 0.80 indicating very good and ≥0.90 indicating excellent internal consistency [22]. Infraclass correlation coefficients (ICCs) were interpreted as indicating moderate reliability (0.50–0.75) and good reliability (0.75–0.90) [23].

A single factor was retained from the original scale after varimax rotation, and construct validity and the underlying factor structure were investigated using an exploratory factor analysis (EFA). Factors with eigenvalues (λ) equal to or greater than 1.0 were retained based on the principal component extraction method [24].

The adequacy of the data for factor analysis was evaluated using Bartlett’s test of sphericity and the Kaiser–Meyer–Olkin (KMO) test. The cut-off values indicating the suitability of the data for factor analysis were set at a KMO value ≥ 0.60 and a Bartlett’s test of sphericity with a significance level of <0.05 [25].

The adapted and original scales’ factor structures were compared using CFA to identify similarities and differences and to evaluate the model’s suitability for the relevant society [26]. Model fit was evaluated using the following indices: chi-square/*df* (χ^2^/*df*), Comparative Fit Index (CFI), Tucker–Lewis Index (TLI), Root Mean Square Error of Approximation (RMSEA), and Standardized Root Mean Square Residual (SRMR) [27]. In evaluating model fit, the following thresholds were considered acceptable: a chi-square to degrees of freedom ratio (χ^2^/*df*) < 3, CFI and TLI values ≥ 0.90, and both SRMR and RMSEA values ≤ 0.08.

Continuous variables were reported as mean (M) and standard deviation (SD), and categorical variables were reported as frequencies (n) and percentages (%). Convergent validity was examined via Pearson correlations between EREC and EDE-Q-13 scores. Multiple regression analyses were performed to determine whether disordered eating characteristics predicted EREC scores. Statistical significance was defined as a *p*-value < 0.05.

## 3. Results

### 3.1. Reliability

An EFA was conducted on a sample of 136 participants to investigate the construct validity of the measure. The mean age was 21.6 ± 2.96 years due to the short sample size. The Kaiser–Meyer–Olkin (KMO) was 0.801, signifying acceptable sample adequacy, while Bartlett’s test of sphericity was significant (χ^2^ = 636.159, *p* < 0.001). Principal component analysis with Varimax rotation revealed a one-factor solution that explained 44.33% of the total variance. This level of explained variance is considered acceptable, as a range between 40% and 60% is deemed sufficient in social science research [24]. Furthermore, all item factor loadings were above 0.50, demonstrating a structure highly consistent with the original scale. Descriptive statistics and item factor loadings are presented in Table 1. With a Cronbach’s alpha of 0.854, the scale’s internal consistency was outstanding. Test–retest reliability was very strong, with an ICC of 0.811 (95% CI: 0.743–0.862).

### 3.2. Construct Validity

**Figure 2:**
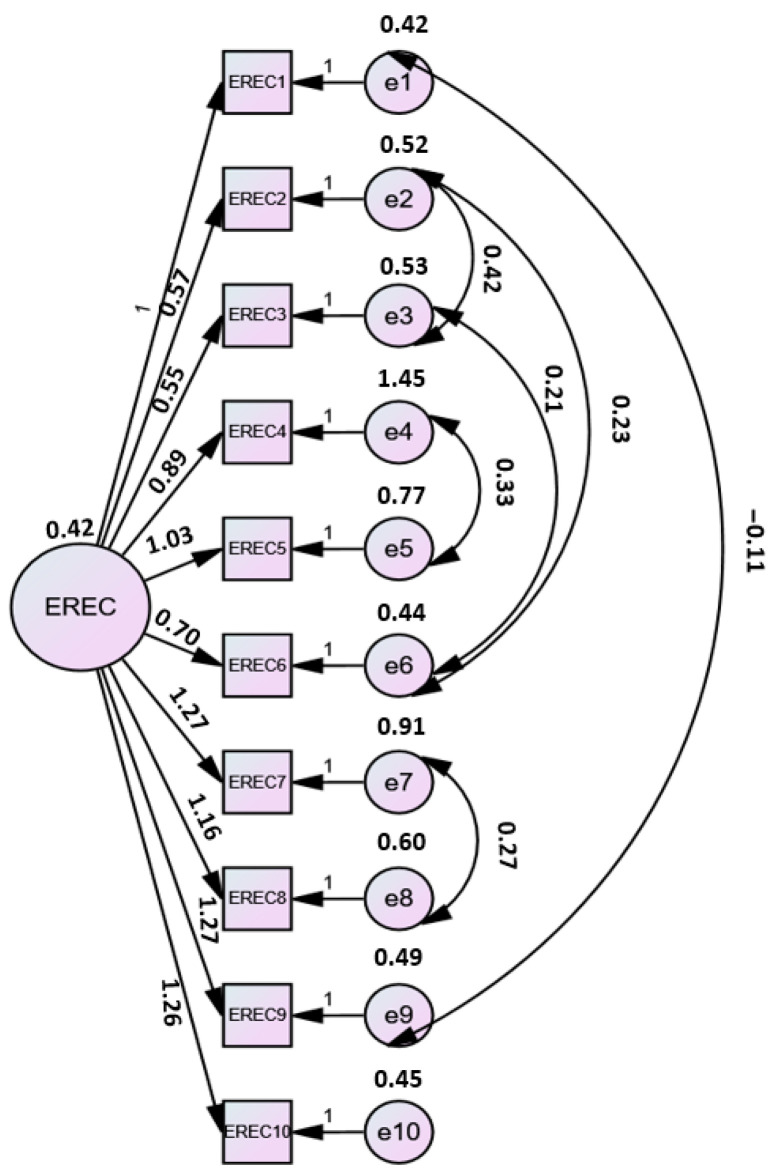
Confirmatory factor analysis model of the Turkish version of the EREC Scale.

A CFA was conducted on the larger sample (n = 464) to assess the scale’s construct validity. The mean age was 21.6 ± 2.48 years, with females constituting 76.7% (n = 356) and males comprising 23.3% (n = 108). The analysis revealed that the single-factor model demonstrated an acceptable fit to the data (χ^2^/*df* = 2.84, CFI = 0.976, TLI = 0.962, RMSEA = 0.063, SRMR = 0.038, AGFI = 0.932) (Figure 2).

### 3.3. Convergent Validity

Table 2 shows the total EDE-Q scores and the scores for the EDE-Q subscales. Eating Restraint, Shape and Weight Over-Evaluation, Binging, Purging, and Body Dissatisfaction are sub-factors of EDE-Q-13. For convergent validity, a small but significant positive correlation was found between EREC and eating restraint (r = 0.199, *p* < 0.001) as well as between EREC and purging behaviors (r = 0.146, *p* = 0.002). Conversely, EREC scores showed no significant associations with shape and weight over-evaluation (r = 0.046, *p* = 0.322), binging (r = 0.025, *p* = 0.596), or body dissatisfaction (r = 0.009, *p* = 0.841). Similarly, there was no statistically significant correlation between EREC and the total EDE-Q-13 score (r = 0.086, *p* = 0.064) (Table 2).

Multiple linear regression analysis was conducted to identify predictors of EREC scores. The model explained 12.7% of the variance in EREC scores (R^2^ = 0.127, *p* < 0.001). Results demonstrated that eating restraint (β = 1.619, *p* < 0.001) and purging behaviors (β = 3.100, *p* < 0.001) were significant positive predictors of EREC scores. Conversely, binging and the over-evaluation of shape and weight were not significant predictors (*p* > 0.05). Notably, the total EDE-Q-13 score was a significant negative predictor of EREC scores (β = −2.648, *p* = 0.028). This shift in the direction of the relationship suggests a suppressor effect; once the specific variances of eating restraint and purging behaviors are controlled for, the residual global severity of disordered eating appears to be inversely associated with eco-concern in eating behavior. This finding suggests that greater severity of disordered eating may be inversely associated with individuals’ eco-concern in eating behavior (Table 3).

## 4. Discussion

### 4.1. Validity of the EREC Scale

The climate crisis, a pressing global problem, has wide-ranging impacts [28]. The recent climate crisis, known as the ecological concern, can have negative effects on people, especially psychologically [5,29]. Since 2017, ecological concerns, which have been heavily featured in visual media, have gained popularity among teenagers and young adults [30,31,32]. An important reflection of ecological concern is also seen in eating behaviors [17]. In recent years, issues related to the climate crisis and environmental sustainability have attracted increasing awareness in Türkiye, particularly among young adults [8]. Within this demographic, the growing sensitivity toward the environmental impacts of food choices—such as water footprint, local production, and waste management—has shifted eating behaviors from being solely health-oriented to encompassing ethical and ecological dimensions [9,31]. The association identified in our study between EREC scores and restrictive eating behaviors among young adults validates the concrete reflections of this societal sensitivity on individual dietary habits. In this study, the validity and reliability of the Turkish version of the “Eating-Related Eco-Concern (EREC)” scale developed by Qi et al. [2] were evaluated among young adults. The scale demonstrated high reliability across both construct validity and internal consistency (Cronbach’s α = 0.854; ICC = 0.811). The results show that the single-factor structure provides adequate model fit and is consistent with the psychometric properties of the scale’s original version. A study conducted in the American population, where the scale was developed, also reported internal consistency (0.88) similar to that of this study [2]. The same scale was also found to be valid and usable in the Italian version [33]. The validity and reliability of the scale were also demonstrated in the Arabic version conducted on Lebanese adults by el Zouki et al. in 2024 [34]. A comparable Cronbach’s α value (0.827) was reported in another study that validated the measure among Turkish adults aged 18–65 [35]. This suggests that many versions of the scale are compatible across cultures and can be used safely to determine a problem that is becoming increasingly widespread worldwide.

### 4.2. The Relationship Between EREC and Disordered Eating Characteristics

Eating behavior problems, which can interact with a wide range of circumstances, are one area in which the EREC scale might be applied. Concerns about climate change can have negative effects, especially on individuals prone to eating disorders [11]. The association between EREC scores and eating disorder symptoms is also investigated in this study. The results demonstrated a substantial positive correlation between eating restraint and eco-concern (*p* < 0.05). Other studies have also shown that individuals may be trained to adopt more restrictive eating behaviors for ecological reasons [32,36]. The general perception is that more environmentally beneficial, sustainable diets are paralleled by lower energy and meat consumption [9,37]. For instance, a study of young individuals discovered a positive correlation between lower meat consumption and higher levels of eco-anxiety [38]. At this point, although behaviors such as “eating less” or “avoiding animal products” begin with motivation to protect the environment, in some individuals, these behaviors can evolve into restrictive habits that carry the risk of an eating disorder. The fact that eco-anxiety produces mental upheaval and unhealthy eating habits may be one of the reasons for this circumstance [39]. In this context, it should be kept in mind that sustainable nutritional practices do not always go hand in hand with healthy eating behaviors and, in some cases, may be confused with pathological tendencies.

Food insecurity, or the difficulty of obtaining food in the future, is one factor contributing to nutrition concerns associated with climate change [40]. A study examining the connection between eating disorders and food insecurity discovered a statistically significant correlation between food insecurity and bulimia nervosa and binge-eating behaviors [41]. In fact, a case study reported the effect of ecological concerns preventing recovery in the treatment of a woman with bulimia nervosa, and it was stated that efforts were made to eliminate this concern as part of the treatment [13]. Eating disorders, eco-anxiety, and associated psychological characteristics among college students are significantly correlated, according to another study. High levels of eco-anxiety were found to be strongly correlated with bulimic eating disorders [12]. In this study, although no significant relationship was found between environmental concern and binge-eating behavior, a significant positive relationship was observed with purging (*p* < 0.05). Although this study did not directly investigate the relationship between eco-anxiety and bulimia nervosa, the frequent occurrence of purging behaviors in bulimia patients raises concerns about a significant relationship between purging and eco-anxiety. Furthermore, it has been suggested that different eating behavior tendencies may be connected to ecological concerns. For example, one study found that eco-guilt and eco-anxiety were associated with orthorexia nervosa and general eating disorder tendency [14]. It is well known that the repercussions of the climate crisis would not only affect the natural world but also people physically and psychologically. Anxiety disorders, mood disorders, acute stress reactions, post-traumatic stress disorder, and aggressiveness are some of these causes. People’s ecological worry over the possible repercussions of climate change is one of these effects. Individuals with ecological anxiety may also exhibit a variety of behavioral, physiological, emotional, and cognitive symptoms [42]. During young adulthood, which is thought to be one of the most vulnerable times for the emergence of eating disorders and mental health syndromes, these symptoms may manifest collectively and severely [43,44]. According to this viewpoint, among the variables to be considered in the development of eating disorders are significant ecological factors that can modify behavior. However, it should not be forgotten that eating behavior disorders are also associated with many individual factors.

### 4.3. The Relationship Between EREC and the EDE-Q-13

According to the study’s findings, eco-anxiety may contribute to some people’s unhealthy eating habits in addition to being a term linked to environmental activism or awareness. Especially in vulnerable groups such as young adults, the psychological motivations underlying sustainability behaviors need to be carefully evaluated. On the other hand, no relationship was found between EREC and EDE-Q-13 total scores in this study. A similar situation was detected in the original study of the scale and in another validity study [2,33]. According to the original study, the EREC scale’s questions are no longer always indicative of an eating disorder. This scale can create awareness of excessive eating behaviors and enable evaluation. Furthermore, it is possible that certain items within the total eating disorder score—perhaps those reflecting general anxiety or obsessive–compulsive traits—may suppress an individual’s eco-concern when they are isolated from concrete behaviors such as restrictive eating and purging. This suggests that while specific pathological behaviors (like purging) are positively linked to eco-anxiety, the broader psychological distress captured by the total score may function differently, potentially masking the environmental motivations behind eating habits.

### 4.4. Strengths and Limitations

It is important to consider some limitations when interpreting the results of this study. First, convenience sampling was used to perform the study among young adults at a particular university, which may limit the data’s applicability to a larger population. Future research involving more diverse age groups and socioeconomic backgrounds would be valuable for validating these findings across a broader demographic. Second, as the data were collected via self-report measures, the potential for social desirability bias should be considered, as participants might have been inclined to underreport certain pathological eating behaviors. Despite these limitations, this study provides a foundational understanding of the relationship between eco-anxiety and eating attitudes.

## 5. Conclusions

This study concludes by demonstrating the validity and reliability of the Turkish version of the EREC Scale. The scale is a suitable tool to assess eating behaviors associated with eco-anxiety among adults. In addition, the findings show that environmental concerns may be linked to eating behaviors in some individuals and that these effects may risk turning into pathological tendencies. Especially in today’s world, where ecological concerns are a major source of anxiety in every area, it is of great importance that healthcare professionals identify individuals who are prone to unhealthy eating habits. These scales can help identify the tendencies of these individuals and enable early intervention. Depending on how common it is in society, it may also lead local or national governments to adopt legislation and conduct preventative measures. However, relatively few studies in the literature have examined ecological issues and their effects on eating habits. More research is required to comprehend the relationship between eating habits and eco-consciousness, given the limited amount of studies.

## Figures and Tables

**Table 1:** Descriptive statistics and factor loadings of the Turkish version of the Eating-Related Eco-Concern (EREC) Scale (n = 136).

Item	M ± SD	Factor Loadings
1. I spend more time than other people searching for sustainable food.	1.8 ± 0.90	0.620 *
2. I avoid eating meat due to concerns about climate change.	1.4 ± 0.83	0.609 *
3. I avoid eating any animal products due to my concerns about climate change.	1.4 ± 0.82	0.582 *
4. I try not to waste food due to concerns about climate change.	3.0 ± 1.38	0.583 *
5. I actively encourage others to change their behaviors to slow climate change.	2.1 ± 1.05	0.694 *
6. I try to eat less because of my concerns about climate change.	1.5 ± 0.76	0.633 *
7. I avoid genetically modified foods due to concerns about biodiversity loss.	2.4 ± 1.22	0.702 *
8. I try to only eat organic foods or food produced without pesticides.	2.2 ± 1.08	0.733 *
9. I avoid foods that come with excess or non-recyclable packaging.	2.3 ± 1.07	0.767 *
10. I pay close attention to information on the impact that certain foods have on the environment (e.g., overfishing, greenhouse gasses, irrigation).	2.2 ± 1.14	0.705 *

M: Mean, SD: Standard Deviation, * *p* < 0.001.

**Table 2:** Pearson correlations between EREC scores and disordered eating characteristics (n = 464).

Disordered Eating Characteristics	M ± SD	r	*p*
Eating Restraint	1.5 ± 1.6	0.199	<0.001 *
Shape and Weight Over-Evaluation	1.9 ± 1.9	0.046	0.322
Binging	0.9 ± 1.2	0.025	0.596
Purging	0.3 ± 0.8	0.146	0.002 *
Body Dissatisfaction	2.1 ± 2.1	0.009	0.841
Total EDE-Q-13 Score	1.3 ± 1.1	0.086	0.064

EDE-Q-13: The Eating Disorder Examination Questionnaire; * *p* < 0.05; r: correlation coefficient 0 < r < 1 is positive corelation, −1 < r < 0 is negative corelation.

**Table 3:** Multiple regression analysis predicting EREC scores from disordered eating characteristics (n = 464).

Predictor	ß	Std. Error	t (*df*)	*p*-Value
Eating Restraint	1.619	0.363	4.458 (1)	<0.001 *
Shape and Weight Over-Evaluation	0.620	0.448	1.383 (1)	0.167
Binging	−0.176	0.412	−0.427 (1)	0.670
Purging	3.100	0.504	6.153 (1)	<0.001 *
Total EDE-Q-13 Score	−2.648	1.197	−2.211	0.028 *

Total Score: Total Eating Disorder Examination Questionnaire Score; * *p* < 0.05; ß: The effect of the variable. A positive value indicates a positive relationship, while a negative value indicates an inverse relationship. t: Significance test of the coefficient. The higher the t-value (positive or negative), the more significant the coefficient. *df*: Degrees of Freedom determines the shape of the t-distribution.

## Data Availability

The data are not publicly available due to restrictions that prevent disclosure of information that could compromise the privacy of research participants.

## Supplementary Materials

The following supporting information can be downloaded at: https://www.mdpi.com/article/10.3390/nu18071132/s1. The survey form used in the study is included as Supplementary Materials File S1: Evaluation of the relationship between eating disorders and food-related eco-anxiety.

## Abbreviations

The following abbreviations are used in this manuscript:

CFA	Confirmatory Factor Analysis
CFI	Comparative Fit Index
EFA	Exploratory Factor Analysis
EREC	The Eating-Related Eco-Concern
EDE-Q-13	The Eating Disorder Examination Questionnaire
ICC	Infraclass correlation coefficient
KMO	The Kaiser–Meyer–Olkin
M	Mean
RMSEA	Root Mean Square Error of Approximation
SD	Standard deviation
SRMR	Standardized Root Mean Square Residual
TLI	Tucker–Lewis Index
