# Supplementary material for: Measuring the Ecological Footprint of Eating Behaviors: A Psychometric Study on the Turkish Version of the EREC Scale
Source: Nutrients. 2026 Mar 31;18(7):1132. doi: 10.3390/nu18071132 (PMC13074659; doi:10.3390/nu18071132)
Supplement: Supplementary file 1 [file nutrients-18-01132-s001.zip › nutrients-4188675-supplementary.pdf]

Full name: Interviewer's name:
Phone number: Questionnaire number:

EVALUATION OF THE RELATIONSHIP BETWEEN EATING DISORDERS AND FOOD-RELATED ECO-ANXIETY

Dear participant,
This research is being conducted to evaluate the relationship between eating disorders and food-related eco-anxiety among young adults. Your data will be used solely for scientific purposes, and your information will remain confidential. Thank you for your interest.

A. GENERAL INFORMATION

**1.** Gender	1.Male	2.Female
**2.** Date of birth:	……./……../……..	
**3.** Marital status	1.Married	2.Single
**4.** The university where you received your education		
**5.** The faculty where you received your education		
**6.** The department you are studying in		
**7.** Your employment status	1. Yes	2. No
**8.** Your place of residence	1. Home alone	2. At home with your friends
	3. At home with your family	4. Dormitory

B. HEALTH INFORMATION AND NUTRITIONAL HABITS

**1.** Do you have a chronic illness(s) diagnosed by a doctor? If yes, what is your illness(s)?		1. Yes	2. No
Cardiovascular diseases		1. Yes	2. No
Diabetes		1. Yes	2. No
Hypertension		1. Yes	2. No
Digestive system diseases (liver, gallbladder, stomach, etc.)		1. Yes	2. No
Respiratory system diseases (lungs, etc.)		1. Yes	2. No
Mental health problems (depression, overeating, vomiting, night eating, etc.)		1. Yes	2. No
Musculoskeletal problems (osteoporosis, joint pain)		1. Yes	2. No
Endocrine (hormonal) diseases		1. Yes	2. No
Vitamin and mineral deficiencies (iron, vitamin B12 and vitamin D deficiency, etc.)		1. Yes	2. No
Neurological diseases (epilepsy, migraine)		1. Yes	2. No
Others		……………………..	
**2.** Are you using vitamin/mineral supplements and/or probiotics?		1. Yes	2. No
**3.** If your answer is "yes," please specify the product(s) you used: ………………		Reason for use	……............
		Usage period	………….
		Usage………number/day/week/month	
**4.** Do you smoke?		1. Yes	2. No
**5.** If your answer is yes, for how many years and how much have you been smoking?		…. year………number/day	
**6.** Do you consume alcohol?		1. Yes	2. No
**7.** If your answer is yes, how often, in what quantity, and which type do you usually prefer?		……………day/week/month<br>…………….ml<br>Drink name ………………	
**8.** How many meals do you usually eat a day?	………Main meal	…… Snack	
**9.** Do you skip main meals? (If not, skip to number 12)	1. Yes	2. No	
**10.** Which main meal do you usually skip?	1. Breakfast	2. Lunch	3. Dinner
**11.** What is your reason for skipping meals?	1.Unable to wake up	2. Not wanting to	3. Habit
	4.Lack of time	5.Lose weight	6.Others
**12.** How many glasses of water do you usually drink?	………… glasses		

C. ANTHROPOMETRIC MEASUREMENTS

**1. Body Weight(kg)**	
**2. Height (cm)**	

D. EATING DISORDER EXAMINATION QUESTIONNAIRE (EDE-Q-13)-SHORT FORM

<u>Explanation</u>

The following questions relate only to the last 4 weeks (28 days). Please read each question carefully. Indicate how many days out of the last 28 days you experienced the following statements. Please answer all questions and select one answer for each question. Thank you.

**EDE-Q-13**	**On how many of the past 28 days ....**	**0**	**1-5**	**6-12**	**13-15**	**16-22**	**23-27**	**Every day**
**1.**	have you been deliberately trying to limit the amount of food you eat to influence your shape or weight (whether or not you have succeeded)?							
**2.**	have you tried to exclude from your diet any foods that you like in order to influence your shape or weight (whether or not you have succeeded)?							
**3.**	have you tried to follow definite rules regarding your eating (for example, a calorie limit) in order to influence your shape or weight (whether or not you have succeeded)?							
**4.**	has your weight influenced how you think about (judge) yourself as a person?							
**5.**	has your shape influenced how you think about (judge) yourself as a person?							
**6.**	have you been dissatisfied your weight?							
**7.**	have you been dissatisfied your shape?							
**8.**	have you eaten what other people would regard as an unusually large amount of food (given the circumstances)?							
**9.**	did you have a sense of having lost control over your eating (at the time that you were eating)?							
**10.**	have such episodes of overeating occurred (i.e. you have eaten an unusually large amount of food and have had a sense of loss of control at the time)?							
**11.**	have you made yourself sick (vomit) as a means of controlling your shape or weight?							
**12.**	have you taken laxatives as a means of controlling your shape or weight?							
**13.**	have you exercised in a “driven” or “compulsive” way as a means of controlling your weight, shape or amount of fat or to burn off calories?							

E. EATING-RELATED ECO-CONCERN QUESTIONNAIRE

Please read each item and indicate how often each statement is true for you. There are no right or wrong answers.

Throughout the PAST MONTH, including today:

		**Nev er**	**Rarely**	**Someti mes**	**Often**	**Always**
**1**	I spend more time than other people searching for sustainable food.					
**2**	I avoid eating meat due to concerns about climate change.					
**3**	I avoid eating any animal products due to my concerns about climate change.					
**4**	I try not to waste food due to concerns about climate change.					
**5**	I actively encourage others to change their behaviors to slow climate change.					
**6**	I try to eat less because of my concerns about climate change.					
**7**	I avoid genetically modified foods due to concerns about biodiversity loss.					
**8**	I try to only eat organic foods or food produced without pesticides.					
**9**	I avoid foods that come with excess or non-recyclable packaging.					
**10**	I pay close attention to information on the impact that certain foods have on the environment (e.g., overfishing, greenhouse gasses, irrigation).					
